# Activation and Pharmacological Regulation of Inflammasomes

**DOI:** 10.3390/biom12071005

**Published:** 2022-07-20

**Authors:** Chen Chen, Pinglong Xu

**Affiliations:** 1 MOE Laboratory of Biosystems Homeostasis and Protection, Zhejiang Provincial Key Laboratory for Cancer Molecular Cell Biology, Life Sciences Institute, Zhejiang University, Hangzhou 310058, China; chenchen1025@zju.edu.cn; 2 Cancer Center, Zhejiang University, Hangzhou 310058, China

**Keywords:** inflammasome, NLRP3, AIM2, caspase, IL-1β, GSDMD, inflammation, targeting, disease, inhibitor

## Abstract

Inflammasomes are intracellular signaling complexes of the innate immune system, which is part of the response to exogenous pathogens or physiological aberration. The multiprotein complexes mainly consist of sensor proteins, adaptors, and pro-caspase-1. The assembly of the inflammasome upon extracellular and intracellular cues drives the activation of caspase-1, which processes pro-inflammatory cytokines IL-1β and IL-18 to maturation and gasdermin-D for pore formation, leading to pyroptosis and cytokine release. Inflammasome signaling functions in numerous infectious or sterile inflammatory diseases, including inherited autoinflammatory diseases, metabolic disorders, cardiovascular diseases, cancers, neurodegenerative disorders, and COVID-19. In this review, we summarized current ideas on the organization and activation of inflammasomes, with details on the molecular mechanisms, regulations, and interventions. The recent developments of pharmacological strategies targeting inflammasomes as disease therapeutics were also covered.

## 1. Introduction

Innate immunity provides the most rapid and conserved defense against cellular damage caused by pathogenic infections, injuries, and cellular stresses. This response depends on the monitoring of pathogen-associated molecular patterns (PAMPs) and damage-associated molecular patterns (DAMPs) through sensors called pattern recognition receptors (PRRs), located in both immune cells and non-immune cells [1,2]. Distinct families of PRRs have been identified, including Toll-like receptors (TLRs) [3], NOD-like receptors (NLRs) [4], C-type lectin receptors (CLRs) [5], absent in melanoma 2 (AIM2)-like receptors (ALRs) [6], retinoic acid-inducible gene I (RIG-I)-like receptors (RLRs) [7], cyclic GMP-AMP synthase (cGAS) [8], and pyrin [9]. Among them, most NLRs and ALRs can form a large protein complex called an inflammasome, which serves as a platform for caspase-1 activation, pro-interleukin-1β (IL-1β) and pro-IL-18 maturation, as well as gasdermin D (GSDMD)-mediated pore formation, or pyroptotic cell death [10,11,12,13]. This review summarizes the recent advances in understanding the mechanisms of inflammasome organization and activation, and the pharmacological approaches that target the inflammasome for therapeutic purposes.

## 2. Discovery and Overview of Inflammasomes

The concept of the inflammasome was first defined by Dr. Jurg Tschopp in 2002 [14], upon identifying a caspase-activation complex consisting of caspase-1, caspase-5, Pycard/ASC, and NALP1 (now known as NLRP1, the nucleotide-binding oligomerization (NOD)−, leucine-rich repeat (LRR)−, and pyrin domain-containing 1), which is responsible for IL-1β maturation. Two years later, Dr. Jurg Tschopp and co-workers discovered the NLRP3 inflammasome and highlighted its essential role in autoinflammatory disorders [15]. Currently, the well-defined inflammasome sensors include the NLR family members NLRP1, NLRP3, and NLRC4 (the nucleotide-binding oligomerization (NOD)−, leucine-rich repeat (LRR)−, and CARD-containing 4), as well as AIM2, and pyrin. The inflammasomes organized by these five sensors can directly engage and activate caspase-1, thus, termed canonical inflammasomes [16]. The canonical inflammasome sensor proteins are widely expressed in many tissues, especially elevated in the bone marrow and lymphoid tissues, and enriched in immune cells such as monocytes, macrophages and neutrophils. Moreover, the NLRP3 inflammasome is reported to be activated in a wide range of endothelial, epithelial, and mesenchymal cells, thus, related to various inflammatory diseases in different organs, such as the skin, brain, heart, and liver [17]. Additionally, the NLRP1 is regarded as the principal inflammasome sensor in human keratinocytes and skin [18]. A variety of inflammasome sensors were identified thereafter, such as CARD8 [19], NLRP2 [20,21,22,23], NLRP6 [24], NLRP7 [25], NLRP12 [26], and interferon gamma-inducible protein 16 (IFI16) [27,28,29], whose mechanisms for inflammasome activation need further exploration.

**Figure 1:**
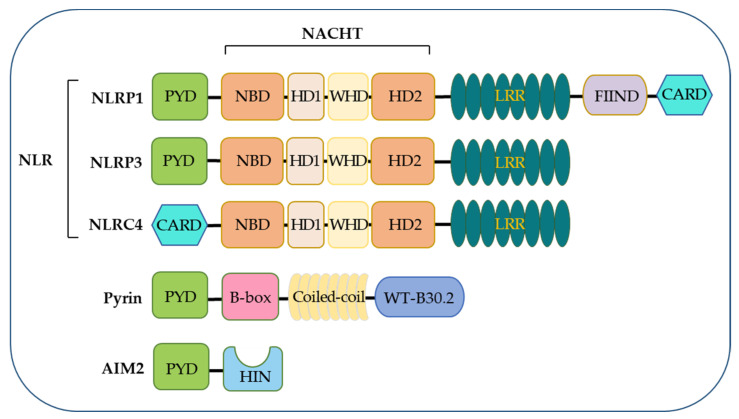
Domain architecture of canonical inflammasome sensors. Overview and alignment of the distinct domains for sensors that initiate canonical inflammasome signaling.

**Figure 2:**
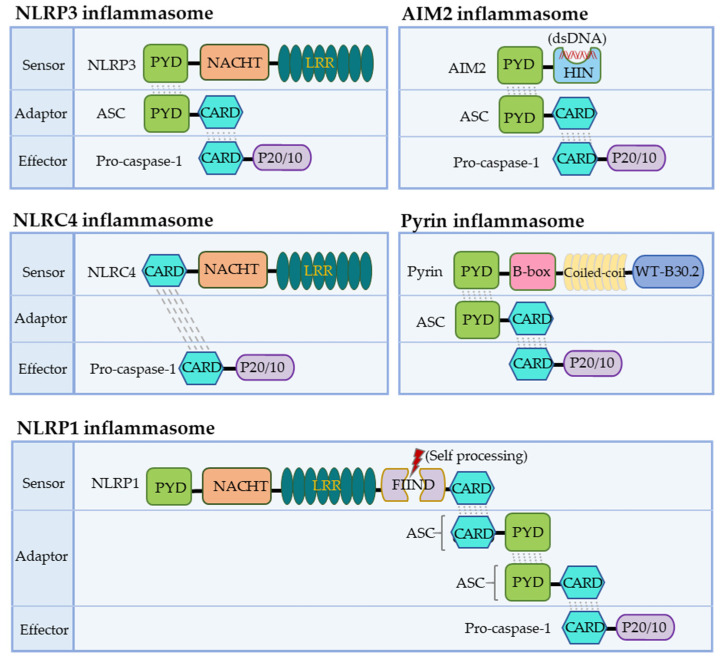
Assembly of canonical inflammasomes. An organization summary for the assembly of canonical inflammasomes, comprising sensors, adaptors/ASC, and effectors/caspase-1.

NLRs function as the primary sensors for inflammasome formation. Structurally, these family members are composed of an N-terminal pyrin domain (PYD), a central nucleotide-binding and oligomerization domain (NACHT), and C-terminal leucine-rich repeats (LRRs). While the NACHT and LRR domains are present in all NLR proteins (except NLRP10), the PYD determining the interaction partners is the most variable (Figure 1). Upon activation, NLR recruits an adaptor protein ASC (apoptosis-associated speck-like protein containing a CARD) through its PYD domain, leading to NLR oligomerization [30]. ASC comprises an N-terminal PYD and a C-terminal CARD domain, which keeps an autoinhibited conformation at the resting state. This pyrin–pyrin interaction allows ASC to release its C-terminal CARD domain, thus, bridging the sensor protein to pro-caspase-1 to assemble a canonical inflammasome via a CARD–CARD interaction [31,32]. Most inflammasomes use ASC to initiate their assembly, while in some particular cases, pro-caspase-1 can be recruited by directly interacting with NLRs through the CARD domain. Pro-caspase-1 is an inactive precursor protein in the resting state. Upon inflammasome assembly, this precursor engages the platform through a CARD–CARD interaction, and the high local concentration of pro-caspase-1 drives its heterodimerization, self-cleavage, and activation [16,33]. Activated caspase-1 converts pro-IL-1β and pro-IL-18 to their mature forms, unleashed into the extracellular and surrounding environment through GSDMD-formed transmembrane pores. These pro-inflammatory cytokines then trigger the activation of NF-κB signaling via transmembrane receptors IL-1R and IL-18R to induce or promote inflammation, locally or distantly [34,35]. The domain organization of NLRP3 and other inflammasomes is illustrated in Figure 2.

**Figure 3:**
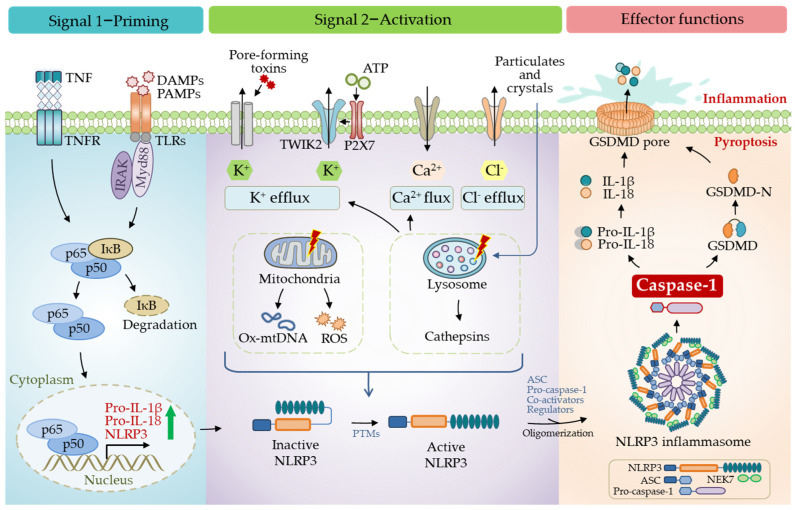
Activation of the NLRP3 inflammasome. Activation of the NLRP3 inflammasome undergoes two sequential steps. The priming process (signal 1) includes the recognition of PAMPs/DAMPs through TLRs and/or sensing of TNFα through TNFR, which induces the expression of NLRP3, pro-IL-1β, and pro-IL-18 proteins via NF-κB signaling. The activation process requires a signal (signal 2) that multiple pathogenic and sterile inflammatory cues can provide. Oligomerization and activation of NLRP3 inflammasome lead to the processing of pro-caspase-1 into the mature form, which in turn processes pro-IL-1β, pro-IL-18, and GSDMD. The released N-terminal domain of GSDMD enters the membrane, forming a pore structure for releasing matured IL-1β and IL-18 into the extracellular space, inducing pyroptosis and causing inflammation.

Another critical and direct substrate of caspase-1 is GSDMD, the primary executor of pyroptosis. Caspase-1 processes cytosolic GSDMD to release its N-terminal domain from an autoinhibited conformation [36,37], a high affinity for the plasma membrane, where they insert and form a pore structure on the membrane [38,39]. This particular structure allows the release of IL-1β and IL-18 into the extracellular space [34,35]. Besides releasing pro-inflammatory cytokines and other contents, plasma membrane pores also cause pathological ion fluxes, ultimately resulting in cell death, known as pyroptosis (Figure 3). A recent study revealed that gasdermin pores are not permanently open, but display phosphoinositide-dependent dynamics, thus, allowing the regulation of cellular pyroptosis [40]. Unlike the canonical inflammasomes that employ caspase-1 as an effector, non-canonical inflammasomes activate caspase-11 in mice and caspase-4/5 in humans [41,42].

This review summarized the recent advances in understanding the organization and activation mechanisms of the inflammasomes, the diseases relevant to their dysfunctions, and the natural and designed inhibitors targeting the inflammasome signaling for a therapeutic purpose.

## 3. Organization of the Canonical Inflammasomes

### 3.1. The NLRP1 Inflammasome

The NLRP1 inflammasome was the first one identified, initially described as a caspase-activating complex comprising caspase-1/5, Pycard/ASC, and NALP1 [14]. Human NLRP1 consists of a PYD, a NOD, a short leucine-rich repeat domain (LRR), a function-to-find domain (FIIND), and a CARD domain (Figure 1). The FIIND domain undergoes a self-processing event before NLRP1 responds to diverse stimuli, a process considered necessary for the complete maturation of NLRP1 [43,44]. Upon activation, human NLRP1 associates with ASC via its C-terminal CARD domain rather than its N-terminal PYD domain (Figure 2). Although NLRP1 contains a CARD domain, the CARD domain of ASC is required and adequate for recruiting pro-caspase-1 via the CARD–CARD interaction [43,44]. Of note, the PYD domain is missing in mouse NLRP1, and the function of human PYD is still elusive. Dipeptidyl peptidases 8 and 9 (DPP8/DPP9) are known as critical inhibitors of NLRP1 [45,46,47], which interact with NLRP1 and, via an unclear mechanism, prevent the proteasomal degradation of its N-terminal fragment [46,48,49]. A recent cryo-EM analysis of the NLRP1–DPP9 complex revealed that the C-terminus of NLRP1 inserts into the tunnel of the DPP9 dimer, thus, advancing the mechanism insight of NLRP1 inflammasome activation [50,51]. Several stimuli have been reported to activate NLRP1 inflammasomes in a protease-dependent manner. Muramyl dipeptide (MDP), a peptidoglycan fragment from both Gram-positive and negative bacteria, directly binds to human NLRP1 and triggers a structural change that promotes NLRP1 oligomerization and assembly [52]. 3C proteases from enteroviruses, such as human rhinovirus (HRV), can cleave human NLRP1 between the E130 and G131 residues to relieve its C-terminal fragment for inflammasome assembly [53]. ORF45 from Kaposi’s sarcoma-associated herpesvirus (KSHV) also facilitates the protease-independent activation of the hNLRP1 inflammasome, which was conserved in primates instead of rodents [54]. Three paralogues of human NLRP1 are found in mice, including NLRP1a, NLRP1b, and NLRP1c. The *Bacillus anthracis* lethal toxin [55], or a reduction in cellular ATP production, results in NLRP1b-dependent inflammasome formation in mice [56]. In contrast to a well-defined NLRP3, the study of NLRP1 inflammasomes is preliminary, both in the activating mechanism and biological relevance. 

### 3.2. The NLRP3 Inflammasome

The NLRP3 inflammasome is the most extensively studied. Human NLRP3 comprises a PYD, a NACHT, and an LRR domain [57]. The NACHT domain, comprising of the nucleotide-binding domain (NBD), helical domains HD1 and HD2, and a middle-winged helix domain (WHD) between HD1 and HD2, is vital for NLRP3 self-association and function (Figure 1). Under resting conditions, the LRR domain folds back onto the NACHT domain and keeps NLRP3 in an ADP-bound, auto-repressed conformation [58]. Once activated, NLRP3 directly binds to mitotic Ser/Thr kinase NEK7 (NIMA-related kinase 7), a recently identified co-activator of NLRP3 [57], and undergoes a conformational transition by hydrolyzing ATP into ADP [59,60]. ATP hydrolysis further triggers the activation of NLRP3, allowing the assembly and recruitment of adaptor protein ASC and pro-caspase-1, thus, enabling caspase-1 self-cleavage and activation (Figure 2 and Figure 3). The assembly of the NLRP3 inflammasome is indicated to occur in mitochondria [61,62,63], as well as in the dispersed trans-Golgi network (dTGN) [64]. Moreover, it has newly been identified to occur at the microtubule-organizing center (MTOC) in an HDAC6-dependent manner, and a similar mechanism has also been observed during pyrin inflammasome activation [65]. The assembly of non-canonical NLRP3 inflammasomes is initiated by caspase-11 in mice, and caspase-4/5 in humans, in response to intracellular LPS, which induces pyroptosis instead of pro-IL-1β and pro-IL-18 maturation. The K^+^ efflux caused by pyroptosis can further trigger the assembly of the NLRP3 inflammasome and promote NLRP3-caspase-1-dependent cytokine maturation [42,66,67]. The NLRP3 inflammasome responds to a surprisingly diverse set of stimuli, the cellular mechanism of which is discussed below (in Section 4. Cellular Mechanisms Driving NLRP3 Inflammasome Activation).

### 3.3. The NLRC4 Inflammasome

NLRC4 was initially identified as a pro-apoptotic protein named the ICE-protease-activating factor (IPAF) based on its similarity to apoptotic protease-activating factor 1 (APAF1) [68]. Besides the NACHT domain and C-terminal LRRs, NLRC4 contains an additional N-terminal CARD domain, enabling the direct recruitment of pro-caspases-1 via a CARD–CARD interaction (Figure 1 and Figure 2). The NLRC4 inflammasome responds to bacterial flagellin and components of the bacterial type III secretory system (T3SS), a process requiring the NLR family of apoptosis inhibitory proteins (NAIPs) as direct upstream receptors [69]. A variety of NAIPs in mice can initiate NLRC4 inflammasome assembly, including NAIP1, NAIP2, and NAIP5/6, which recognize the needle proteins, rod proteins, and flagellin, respectively. However, only one NAIP in humans detects all components [70,71,72]. NLRC4 keeps an auto-inhibited conformation achieved through the inter-domain interaction that covers the nucleotide-binding site [73]. Upon an NAIP recognizing this specific bacterial ligand, the interaction between NAIP and the NACHT domain of NLRC4 triggers the assembly of the NLRC4 inflammasome, followed by the maturation of caspase-1 and the release of inflammatory cytokines and pyroptosis [74,75]. Notably, a NAIP-independent non-canonical NLRC4 inflammasome has recently been identified [76].

### 3.4. The AIM2 Inflammasome

Nucleic acids are predominant PAMPs and ubiquitous in living organisms. In addition to cytosolic DNA sensors such as cGAS and the critical roles of cGAS-STING signaling in cellular physiology [77,78,79,80], AIM2, thus far, is the sole sensor that detects cytosolic double-stranded DNA (dsDNA) among canonical inflammasome sensors [81]. Several independent groups identified AIM2 as a DNA-binding protein while searching for new DNA sensors, and found that it can activate caspase-1 and caspase-3 [82]. The interaction between cytosolic DNA and AIM2 triggers the oligomerization and, ultimately, the formation of the AIM2 inflammasome [82,83]. AIM2 belongs to the AIM2-like receptors (ALRs), structurally defined by two main domains, the HIN domain and the pyrin domain (PYD) (Figure 1). In detecting cytosolic dsDNA, the HIN domain directly binds to dsDNA, thus, relieving the auto-inhibited conformation and releasing the PYD domain, allowing for the recruitment of the adaptor protein ASC and the subsequent AIM2 oligomerization around the DNA molecule. ASC bridges AIM2 to pro-caspase-1 via the CARD–CARD interaction and, in turn, activates caspase-1 and the cleavage of IL-1β and IL-18 (Figure 2). AIM2 is crucial for protecting the host from DNA viruses and other pathogens and the aberrant accumulation of self-DNA in the cytosol. Due to its ability to recognize host DNA, AIM2 involves the pathogenesis of systemic lupus erythematosus (SLE) and tumorigenesis [84]. Intriguingly, recent reports revealed a role of the AIM2 inflammasome in normal brain development through the surveillance of DNA damage during neurodevelopment [85].

### 3.5. The Pyrin Inflammasome

Pyrin (also known as marenostrin and TRIM20) is encoded by the gene MEFV. In 1997, several groups disclosed that the mutations of the MEFV gene in humans are responsible for the monogenic autoinflammatory disease familial Mediterranean fever (FMF) [86]. Later, it proved that the mutations on the C-terminal of human pyrin led to the constitutive activation of the pyrin inflammasome and subsequent caspase-1 activation and IL-1β release [87,88]. Pyrin contains PYD, B-box, and coiled-coil domains (Figure 1). While the PYD domain binds to the ASC through the PYD–PYD interaction, its CARD–CARD interaction with pro-caspase-1 is critical for the recruitment and maturation of caspase-1. The B-box and coiled-coil domain may be involved in the oligomerization of pyrin and modulation of its activation (Figure 2). Unlike mouse pyrin, human pyrin has an additional C-terminal B30.2 domain (also known as a SPRY/PRY), which contains the highest frequency of mutations that lead to FMF. The pyrin inflammasome responds to bacterial toxins and is modified by Rho GTPases. For instance, *Clostridium difficile* TcdB, a major virulence factor of *Clostridium difficile*, covalently modifies RhoA and inactivates these GTPases, which triggers the assembly of pyrin inflammasomes in an ASC-dependent manner [89]. However, pyrin does not directly detect modified RhoA, although many bacterial toxins trigger the pyrin inflammasome through the modification of RhoA [89,90]. Nevertheless, the mechanism by which the pyrin inflammasome is triggered needs further investigation.

## 4. Cellular Mechanisms Driving NLRP3 Inflammasome Activation

### 4.1. The Canonical Pathway

#### 4.1.1. The Role of Priming

Inflammasome activation is generally considered a two-step process, a priming step (signal one) and an activation step (signal two). Initially, the priming step is characterized by the transcriptional induction of NLRP3, pro-IL-1β, and pro-IL-18 through an inflammatory stimulus, including TLR ligands and cytokines such as TNFα [91]. The enhanced expression of these effectors is thought to ensure a rapid and appropriate activation of inflammasomes toward signal two (Figure 3). For instance, LPS is a commonly used ligand to induce the transcription and expression of pro-IL-1β, pro-IL-18, and NLRP3 by activating the transcription factor NF-κB [91]. However, subsequent studies indicated that transcription is dispensable in priming, supported by the time-course observations between the activation of the NLRP3 inflammasome and the upregulation of NLRP3 expression [92,93]. Moreover, although downstream TLR signaling is essential for the rapid NLRP3 activation, this process appears to be independent of the transcriptional function of TLR signaling, but rather depends on adaptor protein MyD88 and the IL-1 receptor-associated kinases IRAK-1 and IRAK-4 [94,95]. Further studies also suggest that post-translational modifications (PTMs) of NLRP3 in the priming process play an essential role in NLRP3 inflammasome assembly, which is discussed later. Collectively, the priming process mainly functions for two purposes: to upregulate the inflammasome components that are ready for a sufficient response to signal two and to order post-translational modifications (PTMs) of NLRP3, which are essential for NLRP3 activation.

#### 4.1.2. Activation of NLRP3 Inflammasome

Multi-faceted pathogenic and sterile inflammatory signals can activate NLRP3, such as foreign PAMPs from fungi, bacteria, and viruses, and host-derived molecules such as reactive oxygen species (ROS), extracellular ATP, and crystalline and particulate matters (uric acid crystals, silica, asbestos, alum, etc.). Additionally, perturbing intracellular homeostasis, including lysosomal destabilization, mitochondrial dysfunction, and ion fluxes (K^+^ efflux, Ca^2+^ signaling, Na^+^ influx, and Cl^−^ efflux), can also activate the NLRP3 inflammasome [96] (Figure 3). The reasons why the NLRP3 inflammasome can respond to such a surprising variety of stimuli are still less known, and, recently, an intriguing report suggests a role of membrane domain disturbance [64].

##### Ion Fluxes

The K^+^ efflux is a joint event during NLRP3 activation, with few exceptions. A decrease in intracellular K^+^ can be induced by ATP and other DAMPs [97,98]. For instance, the purinergic P2X7 receptor (P2X7R) senses the accumulation of extracellular ATP and cooperates with tandem pore domains in weak inward rectifying K^+^ channel 2 (TWIK2), leading to a K^+^ efflux and subsequent NLRP3 activation [99]. Nigericin, a K^+^/H^+^ ionophore, is commonly used as an NLRP3 inflammasome agonist via the K^+^ efflux [97]. Notably, a low extracellular concentration of K^+^ is sufficient to activate the NLRP3 inflammasome, while the high extracellular concentration prevents it [100,101]. A plethora of stimuli, such as LPS, toxins, and particulate matter induce the K^+^ efflux to trigger the NLRP3 inflammasome activation [101]. Despite the vital role of the K^+^ efflux in NLRP3 activation, the exact nature underlying it is still not fully understood.

Calcium-sensing receptor (CaSR) signaling is able to promote NLRP3 inflammasome activation by decreasing the intracellular cyclic AMP (cAMP) level [102,103], as exemplified by platelets that trigger CaSRs signaling and boost NLRP3 inflammasome activation [104]. Additionally, the formation of fetuin-A-based calcium and phosphate-containing matter that functions to prevent extraosseous calcification in vivo increases extracellular Ca^2+^, thus, inducing CaSRs signaling and leading to NLRP3 inflammasome activation in rheumatoid arthritis [105]. ADP released from injured colonic tissue can also activate the NLRP3 inflammasome through P2Y1 receptor-mediated calcium signaling, thereby regulating inflammatory bowel disease [106].

Distinct opinions regarding how the Cl^−^ efflux facilitates NLRP3 inflammasome activation have been presented. While some studies indicated that the Cl^−^ efflux is responsible for the upstream signaling for NLRP3 activation, some groups showed that the Cl^−^ efflux is required for ASC oligomerization [107,108]. The decreased extracellular Cl^−^ level has previously been shown to promote IL-1β secretion [109]. Later, it was found that inflammasome activation led to the translocation of the chloride intracellular channel 1 (CLIC1) and CLIC4 to the plasma membrane, where they mediated the Cl− efflux [107].

Additionally, ion fluxes have frequently been found coordinated in NLRP3 activation. For instance, ATP activates P2X7R, which promotes the Ca^2+^ and Na^+^ influxes and mediates the K^+^ efflux through TWIK2 [99]. The Ca^2+^ flux, regulated by the K^+^ efflux, is presumed to be critical in NLRP3 inflammasome activation [110], although other controversial reports have been presented [111,112]. Therefore, the precise crosstalk among ion fluxes that regulate inflammasome formation remains to be elucidated.

##### Organelle Damage

Studies have shown that particulate stimuli such as alum and cholesterol crystals activate the NLRP3 inflammasome by causing lysosomal rupture and the release of the particulates into the cytoplasm. This notion is supported by dipeptide Leu-Leu-OMe, a soluble lysosomotropic agent, which is sufficient to activate the NLRP3 inflammasome [113]. Similarly, beta-2-microglobulin (beta2m) accumulation in the lysosomes of myeloma-associated macrophages (MAMs) resulted in its disruption, which acted as a driver to initiate NLRP3 inflammation [114]. Additionally, nicotine and carbon-based nanomaterials, such as multi-walled carbon nanotubes (MWCNT), have enhanced NLRP3 inflammasome activation by inducing the lysosomal membrane permeability and releasing cathepsin B [115,116]. Intriguingly, a recent study suggested the direct interaction between cathepsin B and NLRP3 at the endoplasmic reticulum (ER) that facilitates caspase-1 activation [117]. Lysosomal damage is typically accompanied by the K^+^ efflux and Ca^2+^ influx [118], emphasizing its critical role in inflammasome modulation. Lysophosphatidylcholine (LPC), a major lipid component of the plasma membrane, was shown to activate the NLRP3 inflammasome through lysosomal damage and the K^+^ efflux in human monocytes [119].

Additionally, NLRP3 stimuli can promote the disassembly of the trans-Golgi network into the dTGN, which serves as a scaffold for NLRP3 aggregation and activation [64]. Another study also suggested that NLRP3 is translocated to the Golgi apparatus adjacent to a mitochondrial cluster in a complex with sterol regulatory element-binding protein 2 (SREBP2) and SREBP cleavage activation protein (SCAP) to reach optimal assembly and activation [120]. Nevertheless, most current evidence relies heavily on pharmacological approaches that induce ion fluxes and organelle damage simultaneously. Therefore, the exact nature of these events contributing to NLRP3 inflammasome activation needs further investigation.

##### Mitochondria

Besides lysosomes, mitochondria are considered central organelles in regulating the NLRP3 inflammasome activation. Mitochondria can serve as the docking platform for the NLRP3 inflammasome assembly. Upon activation, NLRP3 translocates from the cytosol and ER to the mitochondria and mitochondria-associated membrane, and associates with the adaptor protein ASC [61,62,63]. Several mitochondrial proteins, including cardiolipin, mitofusin 2 (MFN2) [121], mitochondrial uncoupling protein 1 (UCP1) [122], and the adaptor protein mitochondrial anti-viral signaling protein (MAVS), have been reported to interact with NLRP3 and regulate its activation [63].

Small molecules, such as imiquimod and CL097, can drive NLRP3 inflammasome activation by targeting mitochondria and inducing mtROS production [112]. mtROS, released by mitochondria dysfunction, functions as the key upstream signaling in NLRP3 activation. Notably, mtROS-contributed inflammation participates considerably in the progression of Parkinson’s disease [123], cardiovascular risk [124], and human respiratory syncytial virus (RSV) infection [125]. In addition to mtROS, cytosolic mtDNA also mediates NLRP3 activation. A variety of NLRP3 activators can induce mtDNA release, and the synthesis of oxidized mtDNA, following TLR signaling, triggers NLRP3 inflammasome assembly [126]. Moreover, oxidized mtDNA also directly interacts with NLRP3 and functions as a critical component of the NLRP3 inflammasome [127]. During severe fever upon thrombocytopenia syndrome virus (SFTSV) infection, mtDNA is released into the cytosol through BAK/BAX signaling, which activates the NLRP3 inflammasome [128]. Moreover, the mitochondrial electron transport chain (ETC) is essential for NLRP3 inflammasome activation through the phosphocreatine-dependent generation of ATP [129].

##### Co-Activators of NLRP3

Several co-activators of NLRP3 have been identified, including dsDNA-binding protein PKR [130], guanylate-binding protein GBP5 [131], receptor for activated C kinase RACK1 [132], Bruton tyrosine kinase BTK [133,134,135], actin-bundling protein L-plastin [136], stress granule protein DDX3X [137], NLRP11 [138], and NIMA-related kinase NEK7 [139,140,141]. Among them, NEK7 is an essential component of the NLRP3 inflammasome, but not NLRC4 or AIM2 inflammasomes, and the assembly of NEK7 and NLRP3 requires the K^+^ efflux [139]. Recently, the cryo-EM structure of NLRP3-NEK7 was revealed, elucidating an intriguing role of NEK7 by bridging adjacent NLRP3 proteins through the LRR domains of NLRP3, functioning as a licensing step for NLRP3 inflammasome assembly [57]. However, the binding of NEK7 on NLRP3 itself is insufficient to activate the NLRP3 inflammasome. The key factor, which integrates all the signals and directly drives NLRP3 activation, has not been uncovered.

### 4.2. Post-Translational Modifications of NLRP3

#### 4.2.1. Ubiquitination

Emerging evidence in recent years has suggested a critical role of NLRP3 PTMs in inflammasome priming and activation. Ubiquitination-mediated protein degradation controls NLRP3 inflammasome activation by regulating the availability of NLRP3. The lifespan of NLRP3 is coordinated by F-box/LRR-repeat protein 2 (FBXL2) and F-box-only protein (FBXO3) [142]. FBXL2 ubiquitinates NLRP3 and marks its proteasomal degradation, while FBXO3 targets and degrades FBXL2, thus, stabilizing NLRP3 proteins. Besides FBXL2, several other E3 ligases have been identified to mediate the degradation of NLRP3, including tripartite motif-containing protein 31 (TRIM31) [143], Ariadne homolog 2 (ARIH2) [144], casitas-B-lineage lymphoma protein-b (Cbl-b) [145], and membrane-associated RING finger protein 7 (MARCH7) [146]. On the other hand, E3 ligase Cullin1 inactivates NLRP3 through an unknown mechanism, without mediating its degradation [147].

Intriguingly, ubiquitination has a dual role in NLRP3 inflammation activation. The deubiquitination of NLRP3 contributes to inflammasome activation. For instance, the BRCA2-containing complex subunit 3 (BRCC3) deubiquitinase complex interacts with NLRP3 and removes the K63-linked ubiquitin chain from the LRR domain of NLRP3, allowing its oligomerization and activation [148]. By contrast, the inhibition of deubiquitinating enzymes (DUBs), such as ubiquitin-specific peptidase 7 (USP7) and USP47, can completely block NLRP3 activation [149]. Additionally, the stimulator of interferon genes (STING), which acts as an essential adaptor in the DNA-sensing pathway, can promote the localization of NLRP3 in the ER and attenuate its K48- and K63-linked polyubiquitination, thereby enhancing inflammasome activation [150]. By contrast, the E3 ligase Pellino2 and RNF125 [145] positively regulate NLRP3 activation by facilitating the K63-linked ubiquitination of NLRP3 during the LPS-induced priming process. The HUWE1-mediated K27-linked polyubiquitination of NLRP3, NLRC4, and AIM2 promotes their inflammasome assembly [151]. The diverse effects of ubiquitination on the NLRP3 inflammasome probably depend on the modification motifs and cellular localization of NLRP3 and are context-dependent. 

#### 4.2.2. Phosphorylation and Other Modifications

Phosphorylation events control NLRP3 inflammasome assembly and activation, as exemplified by several cases. During the priming, c-Jun N-terminal kinase 1 (JNK1) directly phosphorylates human NLRP3 at Ser198 (mouse Ser194), which is critical for NLRP3 inflammasome assembly and function [152]. The phosphorylation of Thr659 by p21-activated kinases 1 and 2 (Pak1/2) during bacteremia is necessary for the NLRP3–Nek7 interaction, inflammasome activation, and IL-1β maturation [153]. The BTK-mediated tyrosine phosphorylation of the NLRP3 PYD-NACHT linker region promotes inflammasome assembly and IL-1β maturation [154]. Ser295 in human NLRP3 (mouse Ser 293), phosphorylated by protein kinase D (PKD), is essential for inflammasome assembly [155]. However, another group reported that protein kinase A (PKA) could directly phosphorylate the same residue that inhibited the ATPase function of the NACHT domain and, thus, dampened the oligomerization of NLRP3 [156].

Phosphorylation also plays a negative role in regulating NLRP3 activation. For instance, the phosphorylation of human NLRP3 at Ser5 (mouse Ser3) by AKT inhibits NLRP3 inflammasome activation by disrupting the PYD–PYD interaction [157], while protein phosphatase 2A (PP2A) dephosphorylates it [158]. TANK-binding kinase 1 (TBK1) and its homolog, I-kappa-B kinase epsilon (IKKɛ), can also switch off NLRP3 inflammasome activation, although the exact residue(s) modified by these two kinases remains undetermined [159]. Apart from Ser/Thr phosphorylation, the tyrosine phosphorylation of NLRP3 at Tyr918 by Lyn suppresses NLRP3 inflammasome activity [160], while Tyr861 phosphorylation prevents its activation. By contrast, the dephosphorylation of Tyr861 mediated by protein tyrosine phosphatase non-receptor type 22 (PTPN22) relieves this inhibition [161], while the elimination of Tyr32 phosphorylation by PTEN is essential for NLRP3 activation [162].

In recent years, besides ubiquitination and phosphorylation, PTMs have also been shown to emerge in regulating the NLRP3 inflammasome. These NLRP3 modifications include SUMOylation [163,164,165], nitrosylation [166,167,168], alkylation [169,170], acetylation [171], and dicarboxypropylation, which is a rare modification mediated by 4-octyl itaconate, a derivative of immunomodulatory metabolite itaconate [172,173]. Collectively, phosphorylation and various PTMs are critical modulations on the NLRP3 inflammasome, indicating the importance of fine tuning NLRP3 priming and activation.

### 4.3. Non-Canonical Pathway

In addition to the canonical pathway, the NLRP3 inflammasome can be activated via the direct sensing of cytosolic LPS by human caspase-4/5 or mouse caspase-11, the process of which is defined as the non-canonical pathway [41,42,174]. The underlying mechanism of this non-canonical activation is not fully elucidated. Nevertheless, the caspase-11–GSDMD-mediated cellular perturbations may provide a hypothesis in a cell-intrinsic manner. Briefly, intracellular LPS, released by Gram-negative bacteria, can be directly monitored by the CARD domain of caspase-4/5/11, leading to their oligomerization and enzymatic activation through auto-cleavage [42,175]. Subsequently, these activated caspases cleave GSDMD and release their N-terminal cell death domain, which oligomerizes and enters the plasma membrane to form membrane pores and induce pyroptosis [36,37]. The K^+^ efflux caused by GSDMD pores activates NLRP3 and initiates caspase1-dependent IL-1β and IL-18 maturation. Galectin-3 can augment caspase-4/11 oligomerization and promote non-canonical inflammasome activation through LPS binding [176]. Notably, Moretti et al. unveiled another mechanism by which the simultaneous detection of bacterial RNA by NLRP3 and the binding of LPS by pro-caspase-11 promoted a pro-caspase-11–NLRP3 interaction that facilitated their interdependent activation [177]. Besides the intracellular signaling, extracellular LPS activates TLR4 signaling and induces a type I interferon response that upregulates the expression of caspase-11, guanylate-binding proteins (GBPs), and IFN-inducible protein IRGB10 [178,179,180]. GBPs and IRGB10 can lyse intracellular bacteria and release LPS into the cytosol. By contrast, some studies also suggest that extracellular LPS sensing is dispensable for non-canonical NLRP3 inflammasome activation [174,181].

## 5. Roles of Inflammasomes in Diseases

Acute inflammation is beneficial for pathogen clearance and tissue repair, while chronic inflammation is a pathological condition leading to tissue damage. A gain-of-function mutations study on the NLRP3 gene linked its functions to some inherited autoinflammatory diseases [182]. Mutations induce the constitutive activation of the NLRP3 inflammasome and trigger sterile inflammatory diseases, including familial cold autoinflammatory syndrome (FCAS) [182], Muckle–Wells syndrome (MWS) [182], and neonatal-onset multi-systemic inflammatory diseases/chronic infantile neurological cutaneous articular syndrome (NOMID/CINCA) [183]. These three closely related diseases, alternatively called cryopyrin-associated periodic syndromes (CAPS), are characterized by intermittent episodes of a rash, arthralgia, fever, and inflammation in the central nervous system [183]. The increased expression of IL-1β and IL-18 was found in monocytes and macrophages isolated from CAPS patients without any external stimuli [15]. Mechanistically, CAPS-associated NLRP3 mutants form cryo-sensitive aggregates that trigger NLRP3 inflammasome assembly distinct from canonical NLRP3 inflammasome activation [184].

**Figure 4:**
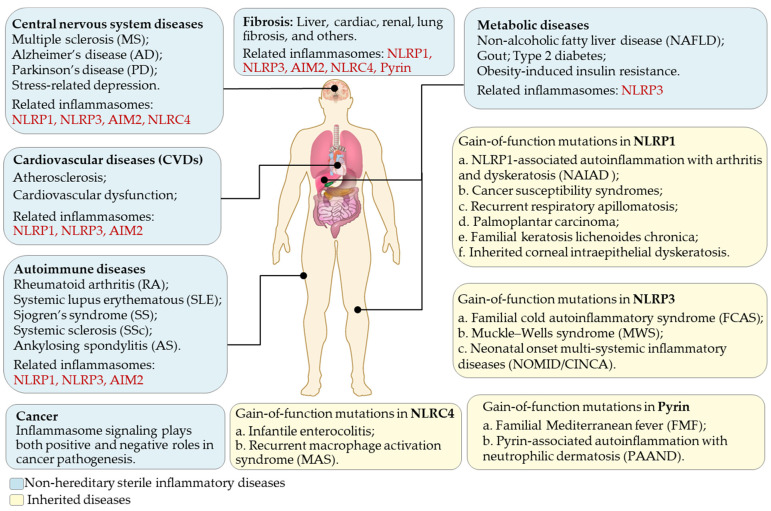
Inflammasome-related human diseases. Canonical inflammasomes are involved in multiple inherited diseases (yellow-shaded boxes) and non-hereditary sterile inflammatory diseases (blue-shaded boxes). The highly relevant inflammasomes responsible for sterile inflammatory diseases are labeled in red.

Besides monocytes and macrophages, the NLRP3 inflammasome can also be activated in a wide range of endothelial, epithelial, and mesenchymal cells, thus, related to various inflammatory diseases in different organs such as the skin, brain, and liver. Thus, the NLRP3 inflammasome has been implicated in central nervous system (CNS) diseases, such as multiple sclerosis (MS), Alzheimer’s disease (AD), and Parkinson’s disease (PD) [185,186], metabolic diseases including non-alcoholic fatty liver disease (NAFLD), gout, type 2 diabetes, and obesity-induced insulin resistance [187], cardiovascular diseases (CVDs) [17,188], and rheumatoid arthritis (RA) [189] (Figure 4). Moreover, the increased IL-1β and ASC expression can be detected in the colon of patients with inflammatory bowel diseases (IBDs) [190], while systemic lupus erythematosus (SLE) patients exhibit an increased level of NLRP3 proteins in macrophages and tissues [191,192]. Evidence also suggests that the NLRP3 inflammasome participates in stress-related depression [193,194], and depressive symptoms can be curbed with anti-inflammatory agents [195]. Recent advances also suggest a role of the NLRP3 inflammasome in ovarian aging and female fertility. An elevated level of NLRP3 proteins was observed in the ovary of female mice during reproductive aging and in granulosa cells from patients with ovarian insufficiency, while the genomic ablation or pharmacological inhibition of NLRP3 ameliorated fertility [196]. Chronic inflammation induced by inflammasomes also contributes to tumorigenesis [197]. By contrast, inflammasome signaling inhibits tumor growth in animal models of colon cancer by maintaining intestinal barrier integrity [198]. Recent studies on Coronavirus Diseases 2019 (COVID-19) showed that the NLRP3 inflammasome contributes to developing respiratory, cardiovascular, and neurological symptoms in COVID-19 patients [199]. Besides the NLRP3 inflammasome, other inflammasomes are also involved in multiple sterile inflammatory diseases (Figure 4). Collectively, inflammasome-mediated inflammation is considerably implicated in a wide variety of human diseases, while manipulating its activation state can be beneficial for treating various diseases. 

## 6. Pharmacological Regulation of Inflammasome

### 6.1. Targeting Inflammasome Assembly

**Figure 5:**
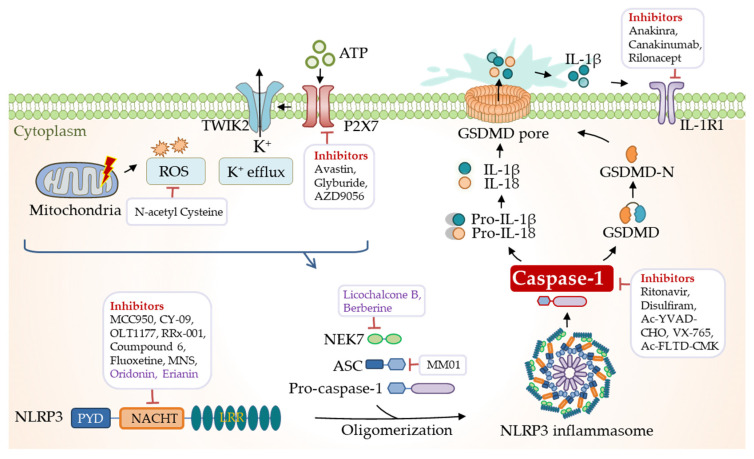
Pharmacological targeting of the NLRP3 inflammasome mechanisms. A wide variety of small synthetic molecules, natural compounds, and antibodies have been developed to target the canonical NLRP3 inflammasome signaling components as research tools and medicines in trials and clinics. Natural, but not synthetic, products are labeled in purple.

NLRP3 is a drug target of significant interest in the pharmaceutical industry, with the potential for treating various inflammatory diseases. Small molecules directly targeting NLRP3 are developing rapidly (Figure 5). The most well-studied one is the diarylsulfonylurea-containing compound, MCC950 (also known as CP-456773) [200], which directly targets the NACHT domain of NLRP3, thus, maintaining NLRP3 in the inactive state [201,202]. MCC950 has been employed as a tool in a wide range of studies on NLRP3-related diseases. For instance, MCC950 was tested in phase II clinical trials for rheumatoid arthritis, but was found to cause liver injuries by elevating the serum liver enzyme levels. Considering the pharmacokinetic and toxic properties, the high doses of the administration of MCC950 were limited in the clinic, and most studies in the field were pre-clinical [203]. MCC950-related compounds, including IZD334 and inzomelid, have completed phase I clinical trials [204]. RRx-001, an anti-cancer agent in phase III clinical trials, is currently identified as a highly selective NLRP3 inhibitor that covalently binds to cysteine 409 of NLRP3 and, therefore, blocks the assembly of the inflammasome [205]. Similarly, fluoxetine, an FDA-approved drug for treating clinical depression, has recently been identified as a direct NLRP3 inhibitor that prevents the NLRP3-ASC assembly [206]. Moreover, MM01 was identified as an inhibitor of ASC that interferes with ASC speck formation [207]. A novel humanized antibody targeting ASC, IC100, was validated to suppress disease in the experimental autoimmune encephalomyelitis (EAE) model [208]. These pharmacological findings provide a new sight into NLRP3-related disease treatment with ensured safety of drugs (Table 1).

### 6.2. Targeting the ATPase Activity of NLRP3

Besides disrupting the NLRP3 inflammasome assembly, many inhibitors are developed to target its ATPase activity (Figure 5). Recently, a series of tetrahydroquinoline inhibitors of NLRP3 inflammasome were discovered, including compound 6 that specifically inhibits NLRP3 activation through directly binding to the NACHT domain and inhibiting its ATPase activity [209], and CY-09 that targets the Walker A motif of the NACHT domain to disrupt ATPase activity [210]. OLT1177 is suggested to covalently modify the NACHT domain to block its ATPase activity [211] and ameliorate cognitive impairment in a mouse model of Alzheimer’s disease [212]. The ATPase activity of NLRP3 can also be interrupted by the direct binding of 3, 4-methylenedioxy-β-nitrostyrene (MNS) [213], and several other compounds, including BOT-4-one [214] and INF39 [215] (Table 1). Nevertheless, the efficacies of these agents in treating NLRP3-driven human diseases need further investigation.

### 6.3. Targeting Upstream and Downstream Signaling

Other alternative strategies have also been attempted to inhibit the NLRP3 inflammasome, such as the blockade of ATP receptor P2X7, NF-κB, caspase-1, IL-1β/IL-1R, and IL-18 (Figure 5). Avastin is a specific inhibitor of the P2X7 receptors (P2X7R), which blocks the ATP-induced activation of the NLRP3 inflammasome [216]. Caspase-1 inhibitors include ritonavir, disulfiram, and VX740/765 [217,218,219], which can inhibit the maturation of IL-1β. Moreover, a GSDMD-derived inhibitor, *N*-acetyl-Phe-Leu-Thr-Asp-chloromethylketone (Ac-FLTD-CMK), was designed to target inflammatory caspase, such as caspase-1, -4, -5, and -11, but not the apoptotic caspases such as caspase-3. It was shown to inhibit GSDMD cleavage and suppress the pyroptosis downstream of both canonical and non-canonical inflammasomes [220]. Notably, the most promising results were found in anti-IL-1β therapy, which is clinically effective in ameliorating inflammasome-related diseases. The FDA has approved three biologics, including the IL-1β receptor antagonist anakinra [221] and the neutralization IL-1β antibodies canakinumab [222] and rilonacept [223]. However, IL-1β signaling is merely one downstream pathway activated by NLRP3, and the complete inhibition of IL-1β signaling may increase the risk of fatal infection in clinical treatment. Intriguingly, the current study indicated that dimethyl fumarate (DMF) could react with GSDMD at its critical cysteine residues to form S-(2-succinyl)-cysteine, therefore, dampening GSDMD-induced cell death [224] (Table 1). These studies provide new strategies to target the elements involved in inflammasome formation and progression.

Other inflammasome sensors, such as NLRP1 and AIM2, are also implicated in diseases (Figure 4). Val-boroPro (VbP, also known as talabostat or PT-100) is an inhibitor of DPP8/DPP9, and has been validated to activate all functional NLRP1 homologs in humans and rodents [45,48,225,226]. Nevertheless, no specific small molecule has, thus far, directly targeted NLRP1 or AIM2 to regulate inflammasomes. The discovery of specific molecule tools for other types of inflammasomes would expand our frontiers in inflammasome biology.

### 6.4. Natural Inflammasome Inhibitors

In addition to synthetic compounds, herb-based medicine shows excellent potential for inflammatory disease treatment (Table 2). An alkaloid from *Piper longum* L., piperlongumine (PL), was shown to disrupt the assembly of NLRP3 and NEK7 and subsequent NLRP3 oligomerization [227]. Licochalcone B (LicoB), a primary component of the traditional medicinal herb licorice, directly binds to NEK7 and inhibits the interaction between NLRP3 and NEK7 [228]. Andrographolide, a bioactive chemical from Andrographis paniculate, is characterized to prevent NLRP3 inflammasome formation [229,230]. Other herb-based medicines identified recently include brevilin A (BA) [231], rpistimerin (Pri) [232], pterostilbene derivatives [233], and berberine [234], which all exhibit protective effects against NLRP3 inflammasome activation (Figure 5). These natural compounds provide potential alternatives in treating inflammatory diseases.

## 7. Concluding Remarks and Perspectives

Inflammasomes are dynamic protein complexes that multiple pathogenic and sterile inflammatory signals can trigger and induce infectious and non-infectious insults. Unlike other innate immune signalosomes that are generally membrane-anchored and less systemic in structure, inflammasomes appear unique. Given the large number of mutually unrelated upstream signals documented, inflammasome sensors seem to respond to general homeostasis stress, but are not a direct agonist. Characterizing the molecular mechanisms underlying inflammasome organization and cellular compartmentation would also be intriguing and critical to understanding this exquisite molecular machinery. Is there a key factor that integrates all the signals? How do the inflammasomes communicate with metabolism and nutrients and form special cellular compartments? Evidently, inhibitors targeting the inflammasome signaling components, such as those developed by studying cGAS-STING signaling [79], provide some attractive potential in understanding the fundamental rationale of this innate immune system and treating various inflammasome-related diseases.

## Figures and Tables

**Table 1:** Small molecule inhibitors targeting NLRP3 inflammasome signaling.

Targets	Agents	Description
NLRP3	Direct	
	MCC950 (CP-456773)	Binds to walker B motif of NACHT domain and locks inactive conformation
	RRx-001	Covalently binds to cysteine 409 of NLRP3
	CY-09	Binds to walker A motif of NACHT domain and inhibits ATPase activity
	Compound 6	Directly binds to the NACHT domain of NLRP3 to inhibit NLRP3 ATPase activity
	OLT1177	Covalently modifies NACHT domain to inhibits its ATPase activity
	MNS	Directly binds to NLRP3, inhibits ATPase activity and prevents NLRP3-ASC interaction
	BOT-4-one	Leads to NLRP3 alkylation, inhibits ATPase activity of NLRP3
	INF39	Inhibits ATPase activity of NLRP3; inhibits priming
	Fluoxetine	Inhibits activation of the NLRP3-ASC inflammasome
	Indirect	
	Thiolutin (THL)	Inhibits NLRP3 deubiquitination and activation
	dihydrotanshinone I (DHT)	Inhibits ASC oligomerization induced by NLRP3 agonists
	methyl gallate	Blocks the ROS over-generation and oligomerization of NLRP3
IL-1β	Canakinumab	Neutralization IL-1β antibody
	Anakinra	IL-1β receptor antagonist
	Rilonacept	Neutralizes circulating IL-1β and IL-1α
P2X7	AZ10606120	P2X7R antagonist
	Avastin	P2X7R inhibitor
	AZD9056	P2X7R inhibitor
	Glyburide	Broad-spectrum inhibitor of P2X7R
Caspase-1	Ritonavir	Specific caspase-1 inhibitor
	Disulfiram	Specific caspase-1 inhibitor
	Ac-YVAD-CHO	Caspase-1 inhibitory peptide
	VX-740/765	Caspase-1 inhibitor
	Pralnacasan (VX-740)	Caspase-1 inhibitor
	Ac-FLTD-CMK	Inflammatory caspases inhibitor, targets caspases-1, -4, -5, and -11
	Tetracycline	Unknown
ASC	MM01	Prevents ASC speck formation
	IC100	Humanized antibody targeting ASC
ROS	*N*-acetyl Cysteine (NAC)	ROS generation inhibitor
NF-κB	BAY-11-7082	NF-κB inihibitor
GSDMD	dimethyl fumarate (DMF)	Reacts with GSDMD and prevents its capacity to induce cell death

**Table 2:** Natural inhibitors targeting NLRP3 inflammasome signaling.

Targets	Agents	Description
NLRP3	Erianin	Directly interacts with NLRP3 and inhibits NLRP3 inflammasome assembly
	Piperlongumine	Inhibits the NLRP3-NEK7 interaction and NLRP3 oligomerization
	Oridonin	Targets Cysteine 279 of NLRP3
	Pristimerin (Pri)	Blocks the assembly of NEK7-NLRP3
	compound 47 (pterostilbene derivatives)	Affects the assembly of the NLRP3 inflammasomes by targeting NLRP3
	Andrographolide	Inhibits the activation of NLRP3 inflammasome
	Tranilast	Binds to NACHT domain and blocks NLRP3-NLRP3 interaction
	β-hydroxybutyrate	Prevents K^+^ efflux and reduces ASC oligomerization and speck formation
	1,2,4-trimethoxybenzene (1,2,4-TTB)	Inhibits the interaction of NLRP3 with ASC
	Parthenolide	ATPase inhibitor and caspase-1 inhibitor
NLRs	Brevilin A (BA)	Inhibits NLRs inflammasomes, blocks ASC oligomerization
NEK7	Berberine	Directly targets the NEK7 protein to block NEK7-NLRP3 assembly
	Licochalcone B (LicoB)	Directly binds to NEK7 and inhibits the interaction between NLRP3 and NEK7

## Data Availability

Data sharing not applicable.
